# Divers' or "Caisson" Disease

**Published:** 1904-06-11

**Authors:** E. H. Snell


### EDITOR'S LETTER-BOX.

[Our Correspondents are reminded that prolixity is a great bar to publication, and that brevity of style and conciseness of statement greatly facilitate early insertion.]

### DIVERS' OR "CAISSON" DISEASE.

*To the Editor of* THE HOSPITAL.

Sir,—In your issue of May 7th there appeared an article on this subject which may lead to some amount of misapprehension. The article refers to the announcement by Professor Leonard Hill *of his discovery* of the mode of causation of this disease by the solution of gases in the blood. In the *Times* of May 14th there appeared a letter of Dr. Hill's in the postcript of which he states that he and Professor Macleod make no claim to this discovery. In Vol. III. of the "Journal of Hygiene," 1903, an article by these writers states that this cause was *proved* to be the true one by Paul Bert's experiments, published in 1878. In 1896 I published a book on "Compressed Air Illness," in which I attempted to show that Paul Bert's explanation was the only one which fully explained the diverse symptoms of this illness. An article by myself, which you were good enough to insert in your issue of May 22nd, 1897, reiterated this. Further, in 1900, a compendious work by Drs. Heller, Mager, and Hermann v. Schrötter, on "Air-pressure Illnesses,' which is the most complete and authoritative book yet published on the subject, puts the matter entirely beyond doubt.

I am not an anti-vivisectionist, but I can agree with your correspondent, Dr. Edward Berdoe, so far as to say that this matter has been so fully determined that further experiments on the same lines as those of Paul Bert and Professor Schrötter have been rendered quite unnecessary.

I am, yours faithfully,

E. H. Snell.

